# Endovascular Abdominal Aortic Aneurysm Repair With Ovation Alto Stent Graft: Protocol for the ALTAIR (ALTo endogrAft Italian Registry) Study

**DOI:** 10.2196/36995

**Published:** 2022-07-11

**Authors:** Gianmarco de Donato, Edoardo Pasqui, Pasqualino Sirignano, Francesco Talarico, Giancarlo Palasciano, Maurizio Taurino, ALTAIR collaborators

**Affiliations:** 1 Department of Medicine, Surgery, and Neuroscience Vascular Surgery Unit University of Siena Siena Italy; 2 Vascular Surgery Unit Sant’Andrea Hospital University La Sapienza Rome Italy; 3 Vascular Surgery Unit Ospedale Civico di Palermo Palermo Italy; 4 See Authors' Contributions

**Keywords:** abdominal aortic aneurysm, endovascular aneurysm repair, endograft, low-profile endograft

## Abstract

**Background:**

Since 2010, the Ovation Abdominal Stent Graft System has offered an innovative sealing option for abdominal aortic aneurysm (AAA) by including a sealing ring filled with polymer 13 mm from the renal arteries. In August 2020, the redesigned Ovation Alto, with a sealing ring 6 mm closer to the top of the fabric, received CE Mark approval.

**Objective:**

This registry study aims to evaluate intraoperative, perioperative, and postoperative results in patients treated by the Alto stent graft (Endologix Inc.) for elective AAA repair in a multicentric consecutive experience.

**Methods:**

All consecutive eligible patients submitted to endovascular aneurysm repair (EVAR) by Alto Endovascular AAA implantation will be included in this analysis. Patients will be submitted to EVAR procedures based on their own preferences, anatomical features, and operators experience. An estimated number of 300 patients submitted to EVAR with Alto stent graft should be enrolled. It is estimated that the inclusion period will be 24 months. The follow-up period is set to be 5 years. Full data sets and cross-sectional images of contrast-enhanced computed tomography scan performed before EVAR, at the first postoperative month, at 24 or 36 months, and at 5-year follow-up interval will be reported in the central database for a centralized core laboratory review of morphological changes. The primary endpoint of the study is to evaluate the technical and clinical success of EVAR with the Alto stent graft in short- (90-day), mid- (1-year), and long-term (5-year) follow-up periods. The following secondary endpoints will be also addressed: operative time; intraoperative radiation exposure; contrast medium usage; AAA sac shrinkage at 12-month and 5-year follow-up; any potential role of patients’ baseline characteristics, valuated on preoperative computed tomography angiographic study, and of device configuration (number of component) in the primary endpoint.

**Results:**

The study is currently in the recruitment phase and the final patient is expected to be treated by the end of 2023 and then followed up for 5 years. A total of 300 patients will be recruited. Analyses will focus on primary and secondary endpoints. Updated results will be shared at 1- and 3-5-year follow-ups.

**Conclusions:**

The results from this registry study could validate the safety and effectiveness of the new design of the Ovation Alto Stent Graft. The technical modifications to the endograft could allow for accommodation of a more comprehensive range of anatomies on-label.

**Trial Registration:**

ClinicalTrials.gov NCT05234892; https://clinicaltrials.gov/ct2/show/NCT05234892

**International Registered Report Identifier (IRRID):**

PRR1-10.2196/36995

## Introduction

### Background

An abdominal aortic aneurysm (AAA) is a dilatation in the lower part of the major vessel (aorta) that supplies blood to the body. The most accepted definition of AAA is based on a diameter of 3.0 cm or more, which is usually higher than 2 SDs above the mean diameter for men [1,2]. Over the last decades, the treatment options have changed. The traditional approach is represented by the open surgical repair [3,4]. As an alternative, the less invasive endovascular treatment has been proposed (endovascular aneurysm repair [EVAR]), which has become the first treatment of choice in patients with suitable anatomy [5,6]. EVAR represents a minimally invasive technique that has overcome some critical issues of open surgical repair such as higher intraoperative and perioperative risk; the necessity of general anesthesia; intensive care unit stay; and higher cardiac, pulmonary, and renal complications [7]. These advantages led to a constant increase in the AAA treatment feasibility, especially in elderly patients with a substantial number of comorbidities that could be treated with reasonable perioperative risks [8] and good early and mid-term outcomes [9] even in emergency settings [10].

Thirty years ago, Juan Parodi [11] developed the first prototype of endograft for EVAR, a handmade device made of a tube-shaped aorto-aortic graft sutured at each end to a balloon-expandable stent based on the design of radiologist Julio Palmaz. This device was implanted in a human body for the first time on September 7, 1990, in Buenos Aires, Argentina [12]. By 1994, the first commercially available device had been launched into the market [13]. Stent-graft material and design changed in various ways to improve conformability, reduce fracture, and minimize device migration rates [14]. Over the years EVAR has become an effective treatment for AAA in patients with challenging anatomy such as hostile neck and small access [15]. Tremendous success has been achieved owing to the continuous technological development that was able to overcome the previous limitations in EVAR applicability. Since 2010, the Ovation Abdominal Stent Graft System (Endologix Inc.) has offered a new concept of sealing, achieved by a network of O-rings filled by a polymer that can treat a great variety of difficult anatomies through a low-profile platform [16]. In the latest version of the stent graft, called Ovation Alto, the conformable O-rings with CustomSeal polymer have been repositioned near the top of the endograft, providing a seal just below the renal arteries. Very few papers highlighting the early and late outcomes of this new device, however, have been published. In this regard, this is intended to be the first multicenter prospective registry study on the implantation of the Alto stent graft in a large cohort of patients, who were also followed up for 5 years with a centralized core laboratory analysis of morphological changes. The aim of this study is to evaluate intraoperative, perioperative, and postoperative results in patients treated by the Alto stent graft (Endologix Inc.) for elective AAA repair in a multicentric consecutive experience.

### Device Under Investigation

**Figure 1:**
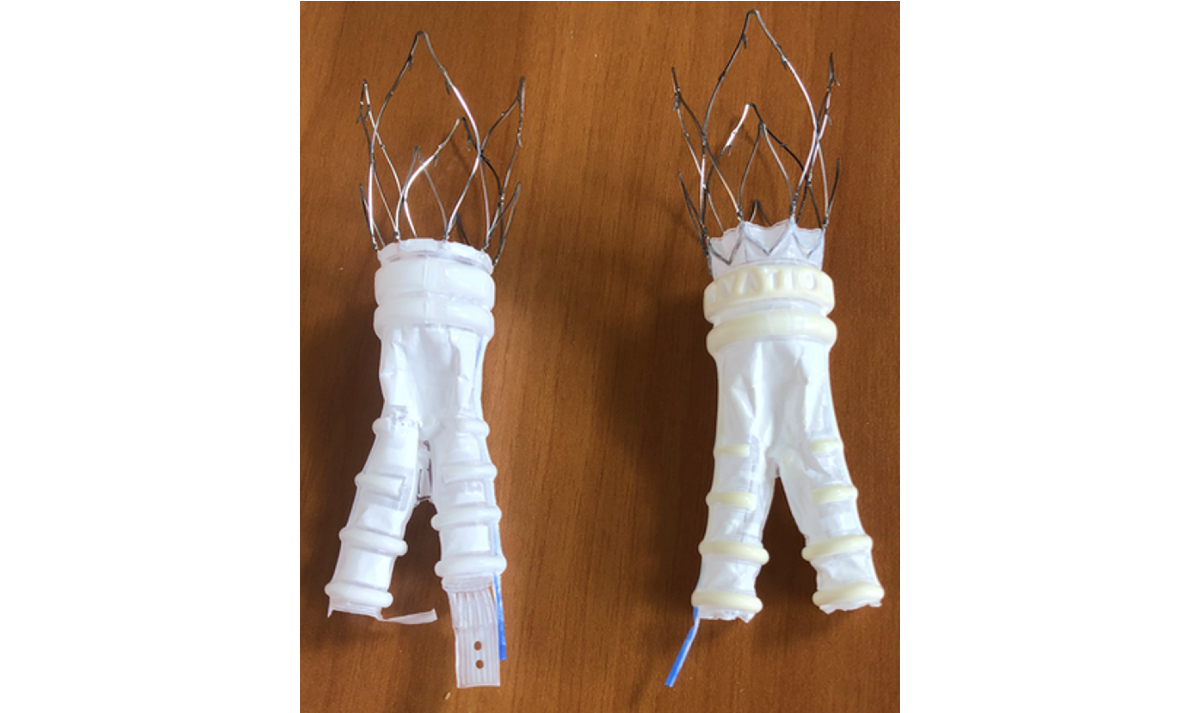
Comparison between Ovation Alto endograft (left) and Ovation iX (right) endograft.

The device under investigation represents the evolution of the low-profile Ovation Prime and iX endograft (Figure 1) [17]. The inflatable channels and the sealing rings, which are the most peculiar features of the predecessor endograft, remained unchanged. This ring network is still filled by a low-viscosity radiopaque polymer intraoperatively, which creates a customized sealing of the infrarenal neck.

Iliac limbs have also not changed and are deployed through a 12-15-Fr delivery system (outer diameter) with various lengths and diameters ranging from 80 to 160 mm and 10 to 28 mm, respectively. The new feature of Ovation Alto is the relocation of the proximal sealing ring at 7 mm from the main body fabric’s proximal edge. In the previous versions, this distance was 13 mm. The low-permeability graft material and the suprarenal 35-mm free-flow stent remained unchanged. It is still delivered via a flexible, hydrophilic-coated, low-profile delivery system (15-Fr outer diameter for all main body measures).

Another design improvement is the integration of a compliant balloon within the delivery system. The balloon is highlighted by a proximal radiopaque marker that coincides with the first sealing ring location, helping in the precise placement of the endograft.

**Figure 2:**
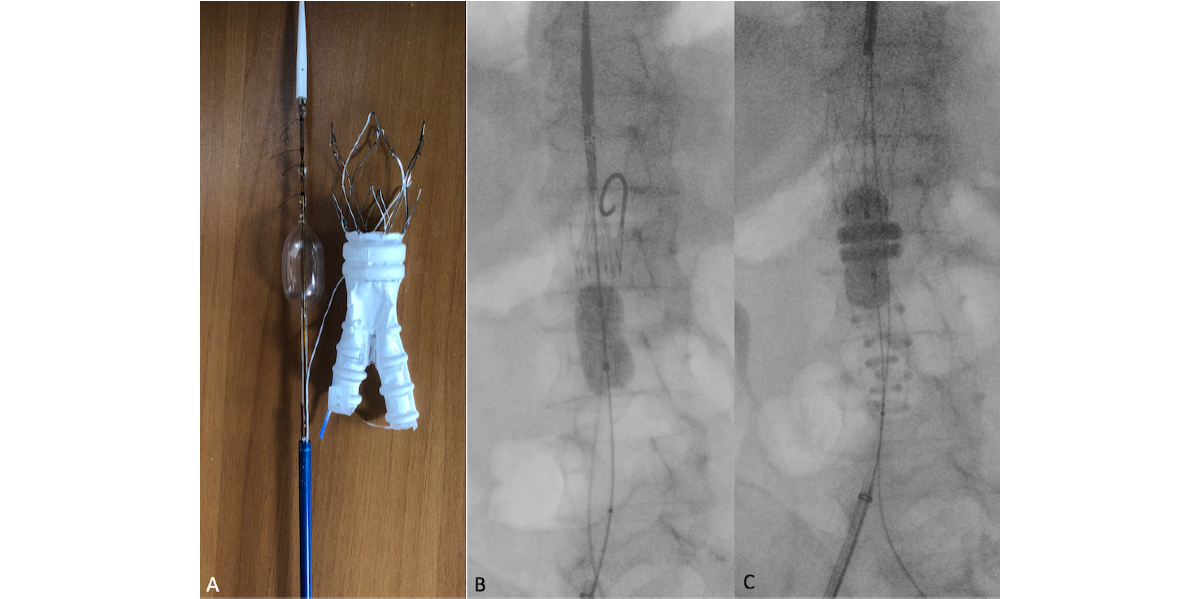
A) Ovation Alto endograft model with the incorporated compliant balloon inflated. B) Intraoperative fluoroscopy with Ovation Alto semi-deployed with the incorporated balloon partially inflated. C) Intraoperative fluoroscopy with Ovation Alto endograft main body deployed, with O-rings polymer filled. The incorporated balloon is advanced of 5-7 mm and fully inflated.

The deployment of the device is performed in a 2-time maneuver (Figure 2). Initially, the lower part of the uncovered stent and the endograft module are deployed and expanded using the integrated, compliant balloon. The upper part of the bare stent remains temporarily undeployed. This system allows the repositioning of the endograft before the final deployment. After full stent-graft positioning and polymer injection, the balloon is advanced by 5-7 mm and re-inflated. This enables a more precise customization of the first sealing ring.

Other minor modifications of the Ovation Alto design are as follows: (1) the inner diameter of the docking limb has been increased to 11 mm for all main body sizing; (2) a more definite web enriched the device bifurcation to avoid a prolapse of the contralateral limb during guide-wire access; (3) the aortic body limbs were offset by 5 mm to improve their identification during the procedure.

According to the current instruction for use, the following anatomical criteria are required [18]:

- adequate iliac/femoral access compatible with vascular access techniques (femoral cutdown or percutaneous), devices, or accessories;
- a proximal aortic landing zone for the sealing ring 7 mm below the inferior renal artery;
- an aortic sealing zone consisting of healthy aorta defined as
  a lack of significant thrombus greater than 8 mm in thickness at any point along the aortic circumference at the level of 7 mm below the inferior renal artery;
  a lack of significant calcification at the level of 7 mm below the inferior renal artery;
  conicity <10% as measured from the inferior renal artery to the aorta 7 mm below the inferior renal artery;
  an inner wall diameter of no less than 16 mm and no greater than 30 mm at 7 mm below the inferior renal artery; and
  an aortic angle of ≤60°.
- a distal iliac landing zone:
  - with a length of at least 10 mm and
  - with an inner wall diameter no less than 8 mm and no greater than 25 mm.

The contraindications for Alto stent-graft implantation according to the instruction for use are:

- patients who have a condition that threatens to infect the graft and
- patients with known sensitivities or allergies to the device materials (including polytetrafluoroethylene, polyethylene glycol–based polymers, contrast agents, fluorinated ethylene propylene, titanium, nickel, platinum, or iridium).

## Methods

### Study Patients

All consecutive eligible patients submitted to EVAR by Alto Endovascular AAA implantation will be included in analysis. Patients will be submitted to EVAR procedures based on their own preferences, anatomical features, and operators experience.

### Recruitment

An estimated number of 300 patients submitted to EVAR with Alto stent graft will be enrolled. It is estimated that the inclusion period will be 24 months. The follow-up period is set to be 5 years. Prior to enrollment into the clinical investigation, patients will be evaluated by their physician for inclusion criteria. Each patient’s medical condition should be stable, with no underlying medical condition that would prevent them from performing the required testing or from completing the study. Patients should be geographically stable, willing and able to cooperate in this clinical study, and remain available for midterm follow-up. Patients who do not wish to participate in this study can obtain the best available EVAR therapy as indicated; refusal to participate in this study will in no way affect their care at the institution. Inclusion and exclusion criteria are detailed in Textbox 1.

This study respects all the principles reported in the current version of Helsinki declaration (2013). AAA morphology will be assessed by OsiriX MD (OsiriX software; PIXMEO) on a computer (with Mac operating system) in a preoperative, contrast-enhanced, computed tomography angiographic (CTA) study. CTA must be performed with a biphasic acquisition protocol (unenhanced and contrast-enhanced scanning with a bolus tracking system) and reconstructions to 1-mm slices. All measurements (diameter, length, and angles) will be evaluated on a workstation with dedicated reconstruction software and center lumen line analysis and multiplanar reconstruction.

**Inclusion and exclusion criteria.**

**Inclusion Criteria**

- Patients with abdominal aortic aneurysm who are scheduled for elective repair according to Endologix Alto endograft device’s instruction for use.
- Patient is willing to comply with specified follow-up evaluations at the specified times for the duration of the study.
- Patient is aged over 18 years.
- Patient, or his/her legal representative, understands the nature of the procedure and provides written informed consent prior to enrollment in the study.

**Exclusion Criteria**

- Abdominal endovascular aneurysm repair performed in an urgent/emergent setting.
- Patients treated outside Endologix Alto endograft device’s instruction for use.
- Patients refusing treatment.
- Patients for whom antiplatelet therapy, anticoagulants, or antihypertensive drug are contraindicated.
- Patients with a history of prior life-threatening contrast medium reaction.
- Life expectancy less than the follow-up period.

A patient is considered enrolled in the study if he/she has full compliance with the study inclusion and exclusion criteria and after successful EVAR procedure at completion angiography.

Clinical data will be collected at patients’ enrollment, EVAR procedure, discharge, planned follow-ups (1-3 and 12 months after the procedure, yearly thereafter), unplanned or interim follow-ups, and patient death. CTA within 90 days, 24 or 36 months, and 5 years after the index procedure is mandatory. Duplex ultrasound scan will be performed at the same follow-up interval, and also at 12, 24, 36, and 48 months. A new CTA will be performed in case of unexpected events during follow-up. The follow-up protocol is based on the most recent European guidelines for the management of the AAAs [1].

### Endpoint

The primary endpoint of the study is to evaluate the technical and clinical success of EVAR with Alto stent graft in short- (90-day), mid- (1-year), and long-term (5-year) follow-up periods. Technical success is defined as the correct graft deployment without any unintentional occlusion of the aortic visceral branches or both hypogastric arteries, with aneurysm exclusion confirmed by the intraoperative angiography, without signs of type I/III endoleak or conversion to open surgery or mortality. Clinical success includes successful deployment of the endovascular device at the intended location without death as a result of aneurysm-related treatment, type I or III endoleak, or graft infection or thrombosis, aneurysm expansion (>5 mm), aneurysm rupture, or conversion to open repair. Moreover, the presence of graft dilatation of 20% or more by diameter, graft migration, or a failure of device integrity will be evaluated.

The clinical and technical successes are defined “assisted” in case of unplanned endovascular procedures, or “secondary” if unplanned surgery is necessary [19].

The following secondary endpoints will be also addressed: (1) operative time; (2) intraoperative radiation exposure; (3) contrast medium usage; (4) AAA sac shrinkage at 12-month and 5-year follow-up; (5) any potential role of patients’ baseline characteristics, valuated on preoperative CTA, and of device configuration (number of component) in primary endpoint.

### Data Collection and Analysis

Patient data will be captured electronically using a computer-based platform accessible to all investigators. Descriptive data summaries will be used to present and summarize the collected data. For categorical variables (eg, gender), frequency distributions and cross tabulations will be given. For numeric variables (eg, patient age), range, mean, median, and SD will be calculated. For all variables, a 95% CI for the relevant parameters of the underlying distribution will be calculated. For all time-dependent events, life tables will be calculated using the Kaplan-Meier estimate method for a period starting on the date of the procedure up to and including all follow-up visits. Stratification to risk factors will be performed and the log-rank test will be used to compare between the different outcomes; associated *P* values <.05 will be defined as significant.

All preoperative and follow-up CTAs were assessed and independently evaluated by 2 experienced vascular surgeons at core laboratory center. Disagreements will be discussed and resolved by consensus.

### Patients’ Confidentiality

All information and data concerning patients or their participation in this clinical investigation will be considered confidential. Only authorized personnel will have access to these confidential files. Authorized personnel of health authorities will have the right to inspect and copy all records pertinent to this clinical investigation. All data used in the analysis and reporting of this clinical investigation will be anonymized.

### Ethical Consideration

This study adheres to the guidelines of European Good Clinical Practice (ICH: 6 R2) and adopted by the Italian Agenzia Italiana del Farmaco (AIFA), in accordance to Legislative Decree 196/2003 and 21/2007 of the Italian Ministry of Health. The local institutional review boards of the participating centers were informed of the descriptive, nonexperimental nature of this registry study. The device under investigation is already available for standard clinical practice.

### Data Availability

The data sets generated during or analyzed during this study will be available from the corresponding author on reasonable request.

## Results

Patient enrollment started in January 2022. It is anticipated that 300 patients will be recruited to the study. All variables will be evaluated in a dedicated central database. All morphological changes will be examined in a centralized core laboratory. After data analysis, results will be shared with each investigator. Updates to results will be published at 1-year follow-up and at 3-5-year follow-up.

## Discussion

The purpose of this registry study is to demonstrate that the adaptive sealing technology of the Alto stent graft is safe and effective in the treatment of infrarenal AAA in different anatomical scenarios.

Since its appearance in the market in 2010, the Ovation endograft has offered an innovative sealing concept involving a nonexpansive circumferential apposition of inflatable rings filled by a low-viscosity polymer [20]. The polymer-filled system adapts to the patients’ aortic neck, thus ensuring a continuous, customized concentric seal. This feature allows a broadening of patient’s eligibility that is higher than other stent graft [21].

The Ovation Alto abdominal stent-graft system received Food and Drug Administration (FDA) approval on March 16, 2020. The commercial launch of the device in the United States was announced on July 30, 2020. On August 5, 2020, Endologix proclaimed CE Mark approval in the European Union for EVAR. In Italy, the commercial launch started in November 2020, in centers having a vast experience with Ovation Prime and iX. The new Alto sealing zone is closer to the renal arteries and may offer sealing in aneurysms with irregular or less than 1-cm long neck. We have already described the possibility to treat juxtarenal aortic aneurysms unfit for open surgery and for fenestrated/branched EVAR. The solution was to perform a physician-modified implantation of Ovation iX, in a procedure termed the “vent” technique. It involves an off-label, aggressive deployment of the sealing ring between 1 and 3 mm below the lowermost renal artery rather than 13 mm. The proximal edge of the collar zone’s fabric was moved down by bare-metal stents contemporarily deployed to assure renal arteries’ patency [22]. The vent technique offered a preliminary evaluation of how the polymer ring may behave close to the renal ostium in very challenging necks, which is now possible with the new design of the Ovation Alto.

An inaccurate deployment of the sealing ring with the previous Ovation version has been described as the cause for an early technical failure [23]. The compliant balloon in the new version of the Alto stent graft may help operators to identify the first ring’s landing, thereby making the deployment more precise. Moreover, the sealing ring’s early ballooning may guarantee a more accurate customization of polymer to aortic wall shape.

The low-profile delivery system (15 F) establishes small iliac access (>6 mm) with the possibility to perform percutaneous procedures, achieving a reduction in blood loss, groin complications, and earlier discharge.

Holden and Lyden [24] reported promising results in the first-in-human experience with the Ovation Alto. At the moment, only early outcomes in a series of 7 patients have been published, while the 5-year results from the Expanding Patient Applicability with Polymer Sealing Ovation Alto Stent Graft (ELEVATE) clinical trial are expected to be published in 2023.

Recently, another initial experience [25] confirmed the early technical and clinical success of the new Ovation Alto stent graft.

Good 5-year EVAR results with the Ovation platform have been reported, demonstrating excellent long-term durability of this endograft, despite 41% of patients having an anatomy unfit for other stent grafts [26]. Results from our registry study may further confirm durability results with the novel version. Moreover, the technical modifications to the endograft may allow for accommodation of a more comprehensive range of anatomies on-label.

## Abbreviations

AAA: abdominal aortic aneurysm
CTA: computed tomography angiography
ELEVATE: Expanding Patient Applicability with Polymer Sealing Ovation Alto Stent Graft
EVAR: endovascular aneurysm repair
FDA: Food and Drug Administration
